# Excitation quenching in chlorophyll–carotenoid antenna systems: ‘coherent’ or ‘incoherent’

**DOI:** 10.1007/s11120-020-00737-8

**Published:** 2020-04-08

**Authors:** Vytautas Balevičius, Christopher D. P. Duffy

**Affiliations:** School of Biological and Chemical Sciences, Queen Mary University of London, Mile End Road, London, E1 4NS UK

**Keywords:** Energy transfer, Light-harvesting, Excitation quenching, Non-photochemical quenching, Photosystem II

## Abstract

Plants possess an essential ability to rapidly down-regulate light-harvesting in response to high light. This photoprotective process involves the formation of energy-*quenching* interactions between the chlorophyll and carotenoid pigments within the antenna of Photosystem II (PSII). The nature of these interactions is currently debated, with, among others, ‘incoherent’ or ‘coherent’ quenching models (or a combination of the two) suggested by a range of time-resolved spectroscopic measurements. In ‘incoherent quenching’, energy is transferred from a chlorophyll to a carotenoid and is dissipated due to the intrinsically short excitation lifetime of the latter. ‘Coherent quenching’ would arise from the quantum mechanical mixing of chlorophyll and carotenoid excited state properties, leading to a reduction in chlorophyll excitation lifetime. The key parameters are the energy gap, $\Updelta \varepsilon =\varepsilon_{\rm Car}-\varepsilon_{\rm Chl},$ and the resonance coupling, *J*, between the two excited states. Coherent quenching will be the dominant process when $-J<\Updelta \varepsilon <J,$ i.e., when the two molecules are resonant, while the quenching will be largely incoherent when $\varepsilon_{\rm Chl}>(\varepsilon_{\rm Car}+J).$ One would expect quenching to be energetically unfavorable for $\varepsilon_{\rm Chl}<(\varepsilon_{\rm Car}-J).$ The actual dynamics of quenching lie somewhere between these limiting regimes and have non-trivial dependencies of both *J* and $\Updelta \varepsilon.$ Using the Hierarchical Equation of Motion (HEOM) formalism we present a detailed theoretical examination of these excitation dynamics and their dependence on slow variations in *J* and $\Updelta \varepsilon.$ We first consider an isolated chlorophyll–carotenoid dimer before embedding it within a PSII antenna sub-unit (LHCII). We show that neither energy transfer, nor the mixing of excited state lifetimes represent unique or necessary pathways for quenching and in fact discussing them as distinct quenching mechanisms is misleading. However, we do show that quenching cannot be switched ‘on’ and ‘off’ by fine tuning of $\Updelta \varepsilon$ around the resonance point, $\Updelta \varepsilon =0.$ Due to the large reorganization energy of the carotenoid excited state, we find that the presence (or absence) of coherent interactions have almost no impact of the dynamics of quenching. Counter-intuitively significant quenching is present even when the carotenoid excited state lies above that of the chlorophyll. We also show that, above a rather small threshold value of $J>10\,\mathrm{cm}^{-1}$quenching becomes less and less sensitive to *J* (since in the window $-J<\Updelta \varepsilon <J$ the overall lifetime is independent of it). The requirement for quenching appear to be only that $J>0.$ Although the coherent/incoherent character of the quenching can vary, the overall kinetics are likely robust with respect to fluctuations in *J* and $\Updelta \varepsilon.$ This may be the basis for previous observations of NPQ with both coherent and incoherent features.

**Electronic supplementary material:**

The online version of this article (10.1007/s11120-020-00737-8) contains supplementary material, which is available to authorized users.

## Introduction

**Fig. 1:**
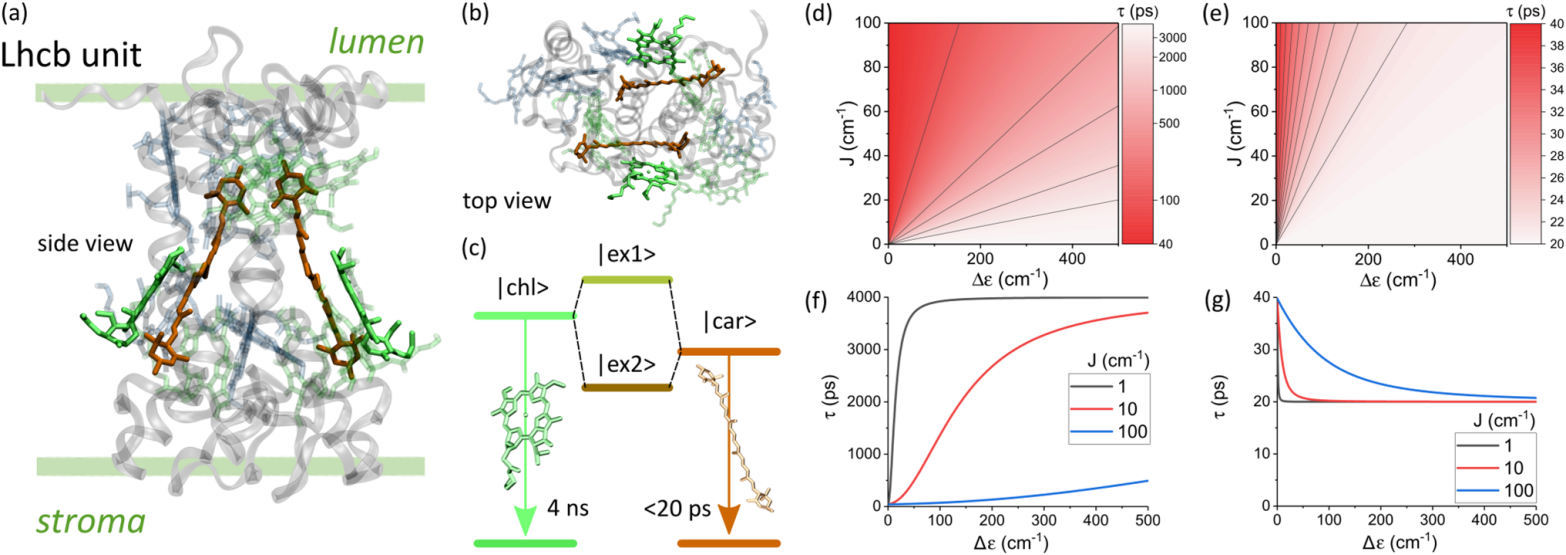
**a** Chlorophyll and lutein mutual geometry within the monomeric LHCII Lhcb sub-unit as seen along the plane of the thylakoid membrane. **b** Car The same as viewed from the stromal side of the membrane (structure taken from Liu et al. 2004). **c** Schematic representation of the excitonic mixing of local molecular states. **d** The lifetime of the Chl-like excitonic state ($$|ex1>)$$ as a function of energy gap, $$\Updelta \varepsilon,$$ and resonance coupling, *J*. The solid lines are contours indicating points where the lifetime of the state is the same. **e** The same for the short-lived Car-like state ($$|ex2>).$$**f**, **g** are a clearer representation of the data in (**d**, **e**) for representative couplings of $$J=1, 10, 100\,\mathrm{cm}^{-1}$$

Quantum coherent energy transfer is now a well-studied concept in the fields of photosynthetic light-harvesting and subsequent charge separation (Scholes et al. 2017). It has been argued that, to a certain extent, coherent transport ensures the remarkable efficiency with which the photosynthetic antennae operate, although this view has been recently challenged (Duan et al. 2017). However, for the survival of plants and their optimal biochemical performance, the down-regulation of the photosynthetic activity under conditions of high illuminations is no less important (Kromdijk et al. 2016; Ruban 2016). While the precise molecular mechanism of such a process, termed non-photochemical quenching (NPQ), is still debated, the involvement of the carotenoid (Car) molecules is widely accepted (Ruban et al. 2012). The most likely candidates for this role are the central lutein (Lut) molecules of the major PSII light-harvesting complex, LHCII, of higher plants and algae (Ruban et al. 2007) (Fig. 1a, b), although a possible additional quenching pathway involving zeaxanthin (Zea) is also being discussed (Leuenberger et al. 2017).

Several quenching mechanisms involving Cars have been proposed. Some postulate that quenching proceeds via the formation of a chlorophyll–Car charge-transfer state which rapidly decays via charge recombination (Holt et al. 2005), while others rely on the intrinsically short lifetime of the lowest singlet excited state of Cars (Ruban et al. 2007). Indeed, excited state lifetimes of chlorophylls (Chl) and Cars, $\tau_{\mathrm{chl}}$ and $\tau_{\mathrm{car}},$ differ by orders of magnitude (see Fig. 1c), meaning that a Car, once brought into contact with a Chl, could possibly act as an energetic ’short-circuit’. Exactly how such a short-circuit would operate is still a matter of debate. One possible scenario is the mechanism of incoherent excitation transfer from the Chl $Q_{y}$ state to the short-lived, optically dark Car state $S_{1}$ (Ruban et al. 2007; Staleva et al. 2015). However, a conceptually different idea of excitonic mixing of lifetimes was also proposed (van Amerongen and van Grondelle 2001). According to this, due to the resonance interaction two delocalized excitonic states are formed by mixing of the $Q_{y}$ and $S_{1}$ ‘local’ molecular states, Fig. 1c. These excitonic states combine the properties of the original local states, including the lifetimes. More precisely, the lifetimes of the mixed states depend on the degree of mixing of the original single-molecule states which in turn depends on their energy difference (the energy gap, here defined $\Updelta \varepsilon =\varepsilon_{\rm Car}-\varepsilon_{\rm Chl}$) and the resonant interaction, *J*, between them. As shown in Fig. 1d–g, the lifetimes of the mixed states are highly sensitive to the changes of these two parameters in certain regions. In particular, the lifetime of the ’Chl-like’ excitonic state, which is purely associated with the Chl in the non-interacting limit, is reduced by orders of magnitude by a small energy gap and/or a large *J* (Fig. 1d, f). It was therefore tempting to speculate that if the LHCII apoprotein under NPQ conditions somehow enforced changes to these parameters [the energy gap often being the preferred one (Liao et al. 2012)], the shortening of the Chl lifetime due to mixing with the Car component could account for the overall reduction of the excitation lifetime within the complex. This reasoning, however, ignores the other ’Car-like’ exctionic state to which the Chl-like state is coupled. The lifetime of the non-interacting Car state is already two orders of magnitude shorter than the Chl-like state and Fig. 1e, f shows that this increases at most by a factor of 2 due to excitonic mixing. It is likely, for certain combinations of $\Updelta \varepsilon$ and *J*, that the overall lifetime of the pair is actually determined by the Car-like state.

The idea of NPQ induction via the excitonic mixing of states gained traction and appeal following the work of Bode et al. (Bode et al. 2009). In this work and several follow-ups (Liao et al. 2010a, b) the two-photon absorption technique was applied to directly access the optically (one-photon) forbidden $S_{1}$ state and a correlation between the onset of NPQ and the Chl–Car interaction parameter was demonstrated. The idea was further studied in artificial Car–phthalocyanine dyads suggesting that the experimentally observed ’bidirectional energy flow’ in the same system [Car to the tetrapyrrole (Kloz et al. 2011) and vice versa (Liao et al. 2011)] is evidence of excitonic coupling (Liao et al. 2012). Additionally, in transient absorption (TA) measurements on certain dyads in specific solvents, induced absorption from $S_{1}$ appeared without a detectable rise time component (Kloz et al. 2011). This, along with the apparent bidirectionality of energy transfer are interpreted as signs of excitonic mixing of the $Q_{y}$ and $S_{1}$ states (Liao et al. 2012). In turn, the correlation between these signs of excitonic mixing and NPQ in isolated antenna complexes or chloroplasts/plants has been interpreted as “excitonic quenching”. A more refined hypothesis proposes that both excitonic mixing and so-called ’bi-directional energy transfer’ contribute to behavior of the quencher (Holleboom and Walla 2014). In this model the energy gap fluctuates about the resonance point. Quenching occurs either incoherently when $\Updelta \varepsilon <-J$ or via lifetime mixing when the fluctuations occasionally bring the two states into resonance. When $\Updelta \varepsilon >+J$ the transfer of energy away from the quencher ($S_{1}\rightarrow Q_{y}$) is assumed to be favored. Rapid fluctuations between $\Updelta \varepsilon <-J$ and $\Updelta \varepsilon >+J$ are said to explain the apparent bidirectionality of Chl-Car energy exchange without the observation of significant mixing of the optical properties of the two states. It was previously proposed that the NPQ switch is some molecular mechanism that controls the equilibrium point, pushing the systems towards $\left\langle \Updelta \varepsilon \right\rangle >+J$ in light-limiting conditions and towards $\left\langle \Updelta \varepsilon \right\rangle <0$ in conditions of excess irradiation (Holleboom and Walla 2014). In 2019 Betke and Lokstein challenged this interpretation proposing that the two-photon absorption of LHCII is actually dominated by Chl $Q_{y}$ rather than Car $S_{1}$ (Betke and Lokstein 2019). However, a more detailed study and a subsequent discussion argued that this is probably not the case (Gacek et al. 2017; Ashfold et al. 2019).

The idea of excitonic mixing playing some essential role in quenching comes with certain issues, both experimental and conceptual. TA experiments on quenched LHCII aggregates revealed fairly slow (~10 ps) energy transfer to the $S_{1}$ state of Lut (Ruban et al. 2007). Our previous theoretical models of the quenched LHCII crystal structure predicted weak resonance couplings between $Q_{y}$ and $S_{1},$ due to the fact that the latter lacks a significant one-electron transition density (Fox et al. 2017; Chmeliov et al. 2015). This was used to justify a purely incoherent model of excitation quenching. While this model did qualitatively reproduce the fluorescence quenching observed in LHCII crystals (Pascal et al. 2005) and aggregates (Ruban et al. 2007) it does not exclude the involvement of excitonic interactions in the real mechanism. More recently Son et al. have shown, via high time-resolution 2D spectroscopy, that the appearance of the Car $S_{1}$ signal in quenched LHCII has a detectable rise time, although it does occur on an ultrafast time scale of $\sim 0.4\,\mathrm{ps}$ (Son et al. 2019), which suggests fairly strong Chl-Car resonance couplings. Although ultrafast energy transfer is not necessarily indicative of excitonic mixing is does indicate that our previous models require revision. Spectroscopic considerations aside, how such an excitonic quencher would act in an actual protein is still an open conceptual question. The original idea of an isolated system of mixed lifetimes (van Amerongen and van Grondelle 2001) completely disregards the energy transfer/redistribution between the excitonic states on the shorter time scales. It was demonstrated that when one takes this redistribution into account, there is no qualitative distinction between the “excitonic” and the incoherent regimes in the long-time kinetics (Balevičius Jr. et al. 2012, 2013). Essentially, it was shown that due to the tremendous difference in excited state lifetimes, the short-lived $S_{1}$ state could be an efficient quencher without mixing of lifetimes, even if it lies energetically above $Q_{y}.$ Lastly, one should keep in mind the fact that even small couplings between localized states can induce considerable quenching of a pigment pool via incoherent transfer (Balevičius Jr. et al. 2017). However, excitonic versus incoherent quenching of excitation in LHCII have in the past been discussed as two functionally distinct and occasionally mutually-exclusive candidate mechanisms for NPQ. Therefore, both the premises of these models and their details in the broader context of excitation dynamics within light-harvesting pigment proteins need to be discussed.

In this paper we explore the idea of excitonic quenching in terms of possible dynamical regimes within the full antenna complex, should such a quenching Chl–Car pair be embedded within it. The key question is whether a coherent quenching mechanism would be functionally or observably different to an incoherent one? We also wish to understand how NPQ fundamentally is switched ‘on’ and ‘off’. Can quenching be substantially altered by changes to the resonance coupling, *J*, the energy gap, $\Updelta \varepsilon,$ or both? Lastly, under excitonic mixing, we should expect both excitonic peak shifts and, in principle, an $S_{1}$ absorption signal which would emerge as it ‘borrows’ oscillator strength from $Q_{y}.$ Therefore we ask whether the presence of an ‘excitonic quencher’ could be, in principle, detectable in the plain linear absorption spectrum of LHCII?

## Model and methods

### Modeling excitation relaxation dynamics within a Chl–Car pair

Energy transfer between two pigment molecules is enabled by their mutual coupling, *J*, while the unidirectionality and irreversibility of such transfer results from the coupling of the pair to their respective surroundings, collectively termed the bath. The ratio of inter-pigment and pigment–bath couplings is to a large degree the decisive factor determining whether the ensuing dynamics can be reasonably described as either coherent or incoherent (May and Kühn 2004; Valkunas et al. 2013). Incoherent energy transfer would generally be expected in the ‘wet and warm’ conditions of biological systems, where the pigment-bath couplings dominate. The dynamics of such a system are well described by the Förster theory of Resonance Energy Transfer (FRET) in which the resonance interaction is treated perturbatively (Förster 1948). In FRET a system of pigments is most intuitively represented in the ’site’ basis, with the excitation stochastically ’hopping’ between localized single-molecule states (the sites). The inverse regime, where the resonance couplings dominate, is realized in systems such as the ring-like LH2 antenna complex from purple bacteria (Papiz et al. 2003). In this case, the appropriate basis is a set of delocalized ’exciton’ states which are quantum mechanical mixtures of the single-molecule states (van Amerongen et al. 2000). In the absence of any coupling to the bath the movement of energy between these exciton states preserves their mutual phase relationships and the dynamics are wave-like or ’coherent’. Coupling of this excitonic system to the bath causes these phase relationships to gradually degrade (in a process known as ’dephasing’), introducing irreversibility and, ultimately, the equilibration of energy across the ladder of excitonic energy levels of the pigment system. For sufficiently weak pigment-bath couplings the dephasing is relatively slow and the dynamics can be described by the Quantum Master Equation (QME) and various derivative approaches such as the well-known Redfield theory (Redfield 1957). The defining feature of QME approaches is the perturbative treatments the pigment-bath interaction [or at least part of it in the case of schemes such as ’modified’ Redfield theory (Zhang et al. 1998)]. It is important to remember the FRET and QME descriptions of the dynamics represent opposing limiting cases. They are convenient approximations based on well-established physical theories but neither is capable of describing the full dynamics in a situation in which resonance couplings and pigment-bath interactions are comparable. It is actually this ’intermediate’ regime that is more typical of photosynthetic light-harvesting complexes.

In this study we employ the so-called Hierarchical Equations Of Motion (HEOM) methodology to describe the dynamics of our Chl *a-*Lut quencher (Ishizaki and Tanimura 2005). HEOM treats the pigment-bath interaction in a non-perturbative manner meaning that it enters the theory on an equal footing to the pigment-pigment couplings. For a given model of the pigment-bath interaction it represents an essentially exact description of the dynamics of the system from which the FRET and QME descriptions emerge as limiting cases. The exact form of the HEOM depends on the specific problem being studied. For a detailed technical analysis of the HEOM description of a general coupled pigment pair the reader is directed to (Balevičius Jr. et al. 2012, 2013). The model presented here is essentially identical but for the fine tuning of some of the parameters to describe specifically a Chl *a-*Lut quenching pair and the embedding of this pair within the wider LHCII complex. For comparative purposes we also model the system in the FRET framework which we employed in our previous models of quenching in LHCII (Balevičius Jr. et al. 2017; Chmeliov et al. 2015; Fox et al. 2017). The mathematical details of these models are presented in the Supplementary Material.

In the following we will denote the first singlet excited states of Chl, $Q_{y},$ and Lut, $S_{1},$ by $|\mathrm{chl}\rangle$ and $|\mathrm{car}\rangle,$ respectively. These two states comprise our ‘site basis’. In the presence of a resonance interaction these states mix and the more applicable representation is the ’exciton basis’,

1 $$\begin{aligned} |a\rangle &= \cos \theta |\mathrm{chl}\rangle +\sin \theta |\mathrm{car}\rangle; \\ |b\rangle &= -\sin \theta |\mathrm{chl}\rangle +\cos \theta |\mathrm{car}\rangle , \end{aligned}$$

where, the parameter $\theta \in [-\pi /4;\,\pi /4]$ is the so-called mixing angle, defined as,

2 $$\theta =\frac{1}{2}\arctan \frac{2J}{\Updelta \varepsilon }.$$

The degree of mixing is (in princple) reflected in the absorption spectrum of the pigment pair as peak shifts (’excitonic splitting’) and the redistribution of oscillator strength (via mixing of the transition dipole moments of the component states) (Valkunas et al. 2013). However, the dynamics of the subsequent energy transfer and dissipation are substantially less trivial to characterize (Balevičius Jr. et al. 2012). Fortunately, HEOM allows for a complete picture of these dynamics, incorporating the effects of excitonic mixing and dephasing implicitly. We are free to represent these dynamics in either the site or exciton basis although, depending on the value of $2J/\Updelta \varepsilon,$ one will be more intuitive than the other. When $2J/\Updelta \varepsilon \rightarrow 0$ state mixing vanishes, the two representations become equivalent (the site basis), while when $2J/\Updelta \varepsilon$ becomes large state mixing is significant and the exciton basis is by far the more intuitive representation. Throughout this work when there is a significant degree of state mixing (typically when $|\Updelta \varepsilon |<J$) we label the resulting dynamics as ’excitonic’. Outside this region of the parameter space we refer to the resulting dynamics as ’incoherent’. It is important to realize that there is no sharp boundary between these two cases and they do not represent two distinct mechanisms. These are simply intuitive approximate descriptions of the real non-trivial dynamcis. We make one explicit addition to our HEOM description. Namely, the mixing of the intrinsic excited state lifetimes of $|\mathrm{chl}\rangle$ and $|\mathrm{car}\rangle.$ The intrinsic decay rates of each state, $\tau_{\mathrm{chl}}^{-1}$ and $\tau_{\mathrm{car}}^{-1},$ transform into the decay rates, $\kappa_{a/b},$ of the excitonic states as (Balevičius Jr. et al. 2012):

3 $$\begin{aligned} \kappa_{a} &= tau_{\mathrm{chl}}^{-1}\cos ^{2}\theta +\tau_{\mathrm{car}}^{-1}\sin ^{2}\theta;\\ \kappa_{b} &= \tau_{\mathrm{car}}^{-1}\cos ^{2}\theta +\tau_{\mathrm{chl}}^{-1}\sin ^{2}\theta. \end{aligned}$$

The intrinsic lifetimes, as in previous theoretical studies, are standard values taken from experiment with $\tau_{\mathrm{chl}}^{-1}=4\,\mathrm{ns}$ and $\tau_{\mathrm{car}}^{-1}=20\,\mathrm{ps}.$

Lastly, it is important to stress the methodological differences between FRET theory and HEOM, and how the results of the two theories should be compared. In principle there are two major issues to be considered: the difference in outputs and their relative capabilities for treating different models of pigment–bath interaction. Let us start with the first issue. Conceptually, the excitation equilibration dynamics within an isolated dimer are characterized by three related time scales: the forward and backward excitation hopping times (the inverse of the forward and backward transfer rate constants, $k_{a\rightarrow b}$ and $k_{b\rightarrow a}$) and the overall thermalization time. The later is the exponential time constant for the equilibration of energy across the two levels and is simply given by $\tau_{therm}=(k_{a-b}+k_{b-a})^{-1}.$ We note that whereas FRET theory explicitly yields the transfer rates (equivalently the hopping times), HEOM provides the evolution of populations and coherences between $|\mathrm{chl}\rangle$ and $|\mathrm{car}\rangle$ states (or $|a\rangle$ and $|b\rangle$). Hence, in order to compare the two methods and, especially, identify any deviations of the dynamics from the standard incoherent FRET regime, we have to extract rate constants from the HEOM population evolutions. We use simple exponential fitting to first determine the thermalization time. The forward and backward transfer rates can in turn be determined from the thermalization time given the detailed balance condition: Namely, that the ratio of the forward/backward rates is equal to the ratio of the equilibrium populations. Additionally, we will use the following convention in this paper: since Chl is considered to be the initial excitation energy donor, we will refer to the Chl-to-Car transfer as ‘forward transfer’. By contrast, transfer from Car, which is the acceptor (and eventually the quencher), is referred to as ‘back-transfer’. In the case of excitonic states, the forward transfer refers to the direction from the state with mostly Chl character to the state with mostly Car character (although this distinction becomes meaningless at the resonance condition, $\Updelta \varepsilon =0$).

**Fig. 2:**
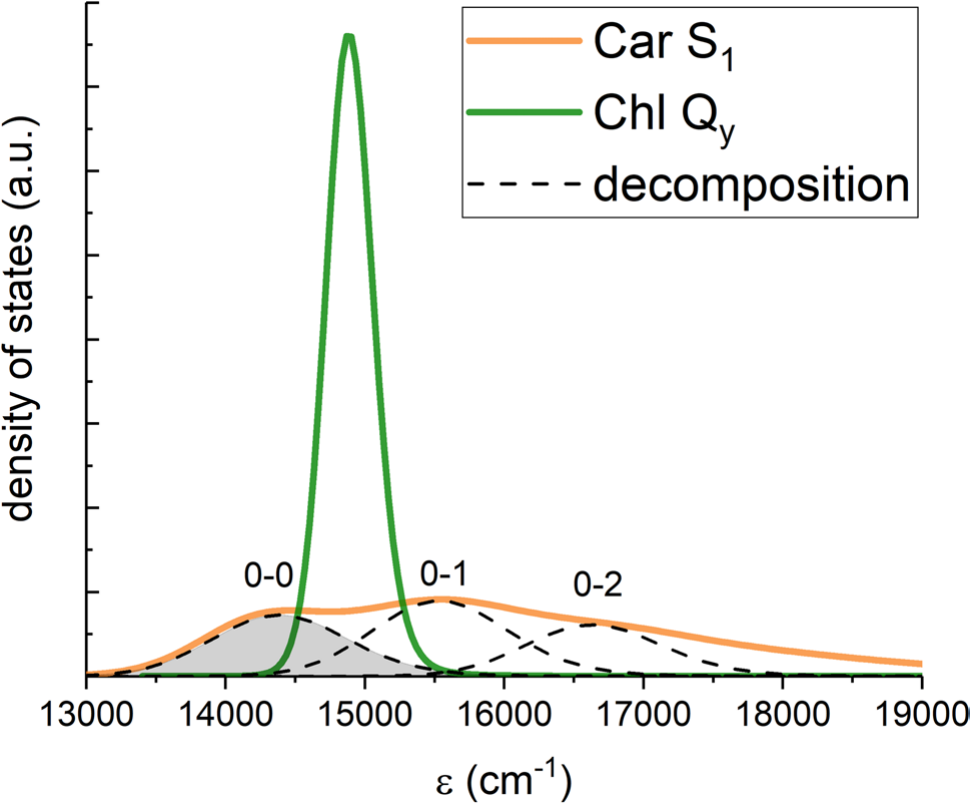
The ’Density of States’ distributions for the $Q_{y}$ (green) and $S_{1}$ (orange) transitions of Chl and Lut, respectively. For the optically-allowed $Q_{y}$ transition (but not for optically-forbidden $S_{1}$) this represents real absorption line-shape. The dashed lines represent the decomposition of the $S_{1}$ density of states into three separate vibronic peaks. The difference in amplitude is due to both functions being normalized

Concerning how FRET and HEOM deal with the pigment-bath interaction the key concept in both cases is the spectral density of the bath, $C^{\prime \prime}(\omega ),$ i.e., the frequency distribution of the vibrational states of the bath, weighted by their strength of interaction with the pigment states. While the spectral density of $|\mathrm{chl}\rangle$ is readily extracted from optical measurements (Renger and Müh 2013), this is not the case for $|\mathrm{car}\rangle$ since it optically-forbidden. However, it is known that the absorption of the optically bright Car $S_{2}$ state is characterized by a vibronic progression of the carbon–carbon single- (C-C) and double-bond (C=C) stretching modes (Polívka and Sundström 2004), and the corresponding spectrum can be accurately reproduced using a spectral density consisting of two underdamped modes, explicitly representing C–C and C=C stretching, and a single overdamped mode implicitly representing the rest of the bath. Earlier we have applied a similar composite spectral density to visually fit the Car $S_{1}$ two-photon absorption spectrum (Fox et al. 2017) reported for Lut in octanol (Walla et al. 2002). The spectrum constructed from the extracted spectral density is shown in orange in Fig. 2. FRET easily accommodates any form of spectral density. For HEOM, while a highly-structured spectral density is in principle possible (Tanimura 2012; Liu et al. 2014), it comes at great computational cost (Kreisbeck et al. 2014). In this work we use the following approach to overcome this difficulty. Since we are primarily interested in the effects of $|\mathrm{chl}\rangle$ approaching resonance with *any* vibronic level on $|\mathrm{car}\rangle,$ we can focus on just a single such level to which we assign a simple unstructured spectral density. In fact, the full absorption spectrum of Car can be viewed as a progression of individual vibronic transitions as shown in Fig. 2 by dashed contours. We can therefore make this simplification without loss of generality. If we were to scan the $Q_{y}$ peak across the entire $S_{1}$spectrum it would sequentially encounter resonance with the three $S_{1}$ vibronic peaks (0-0, 0-1 and 0-2). The qualitative effects within the vicinity of each resonance would be essentially identical. Quantitatively the rates would of course differ because the decomposition of the spectrum is not represented by a decomposition of spectral density. However, the rates should still be similar for the first one or two vibronic transitions. In this work we employ the familiar overdamped Brownian oscillator spectral density (Mukamel 1995) (OBO; also known as Drude model) for individual vibronic levels. It is given by the equation,

4 $$C^{\prime \prime}(\omega )=\frac{{2\lambda \omega \gamma }}{(\omega ^{2}+\gamma ^{2})},$$

where $\lambda$ is the reorganization energy (in this work we use the convention $\hbar =1$), which represents the pigment–bath coupling strength, and $\gamma$ governs the overall shape of the $C^{\prime \prime}(\omega )$ distribution. For Chl *a* we use parameters $\lambda =85\,\mathrm{cm}^{-1}$ and $\gamma =53\,\mathrm{cm}^{-1}$ (Wu et al. 2015), for a single vibronic level of $S_{1}$ we use $\lambda =600\,\mathrm{cm}^{-1}$ to approximately cover the same window of energies as a single vibronic peak in the density of states (the decompostion of the full $S_{1}$two-photon spectrum (orange) is shown in dotted line in Fig. 2), and $\gamma$ identical to Chl *a* (shown in green in Fig. 2). One of the most important observations is that the reorganization energies associated with $S_{1}$ are an order of magnitude larger than for $Q_{y}.$ This is the cause of the very broad two-photon absorption profile and implies a large displacement between $S_{1}$and the ground state. This large displacement has recently been proposed to be a contributing factor the optically-forbidden nature of $S_{1}$ (Fiedor et al. 2019).

### Excitation lifetimes and dynamics within a coupled system of pigments

In order to asses the efficiency of quenching within the dimer and LHCII a suitable measure is needed. Here, as previously (Chmeliov et al. 2015; Fox et al. 2017, 2018; Balevičius Jr. et al. 2017), we consider the ’mean excitation lifetime’, $\tau_{\mathrm{exc}}.$ This is an observable quantitiy (through fluorescence lifetime/yield measurements) and is therefore basis-independant. We can estimate $\tau_{\mathrm{exc}}$ from the evolution of the populations of the system states. In the purely incoherent regime the system dynamics are governed by the Pauli master equation,

5 $$\frac{\mathrm{d}}{\mathrm{d}t}P\left( t\right) =KP\left( t\right),$$

where *P*(*t*) is the vector of populations of the system states (in whichever basis is convenient) and *K* is the rate matrix. The solution to this equation can be obtained by diagonalizing the rate matrix as $K=C\lambda C^{-1},$ where $\lambda$ is the diagonal matrix of eigenvalues, and *C* is the diagonalizing matrix. In this case the solution reads: $P(t)=C\mathrm{e}^{\lambda t}C^{-1}P(0).$ In this study we focus on systems with finite excited state lifetimes, without explicitly including the ground state (for reasons of computational tractability), hence the rate matrix *K* includes additional decay rates $\tau_{\mathrm{chl}}^{-1}/\tau_{\mathrm{car}}^{-1}$ (or $\kappa,$ Eq. 3). Our measure of dissipation efficiency in the complex system described by Eq. 5 is the sum over eigenvalues weighted by the initial populations:

6 $$\tau_{\mathrm{exc}}=-\sum_{i}\left[ \lambda ^{-1}C^{-1}P(0)C\right]_{i}.$$

For the dimer the rate matrix is defined,

7 $$\begin{aligned} K=\left( \begin{array}{cc} -k_{a\rightarrow b}-\kappa_{a} &{} k_{b\rightarrow a}\\ k_{a\rightarrow b} &{} -k_{b\rightarrow a}-\kappa_{b} \end{array}\right) , \end{aligned}$$

where the rate constants are obtained explicitly in the case of FRET and from fitting of the population dynamics in the case of HEOM. We may further extend the description to the full pigment pool of the monomermic (Lhcb) sub-unit of LHCII. In the latter case, the rate matrix *K* for the pool of 14 Chl (8 Chl *a* and 6 Chl *b*) has been presented earlier (Chmeliov et al. 2015). We employ the same rate matrix, but append the additional decay rates due to the addition of two Luts, and so our *K* matrix ($16\times 16$) now reads:8Firstly, we have assumed that their are two quenching pairs within the sub-unit. These are the centrally bound Luts L1 and L2 which are coupled repspectively to Chl a612 and Chl a603 (in the notation of Liu et al. (2004)) which we here label ’3’ and ’12’. We have previously shown that L1 and L2 have very limited interactions with the other Chls Chmeliov et al. (2015); Fox et al. (2017). We ignore violaxanthin (Vio) and neoxanthin (Neo) as the former does not couple to the Chls and the latter couples primarily to Chl *b* (Fox et al. 2017). We note that the labels L1, L2, 3, and 12 are only strictly applicable in the in the absence of excitonic mixing. However, we more generally use L1 and L2 to represents the two Car-like mixed states in each pair and 3 and 12 to for the Chl-like states (although this distinction becomes meaningless at the resonance point). Similarly $k_{12\rightarrow L2},$ etc. represents the rate constant extracted from FRET or the HEOM evolutions. Lastly, the dotted box represents the ($14\times 14$) *K* matrix of the Chl pool only, which was calculated previously (Chmeliov et al. 2015) and ${\mathbf {0}}$ is used to denote a matrix block of zeroes.

### The linear absorption spectrum of an LHCII monomer

We also examine whether excitonic quenching could possibly manifest (as changes in peak position and intensity) in the absorption spectrum of the LHCII monomer. The spectra were calculated using the method described in (Bašinskaite et al. 2014), starting with stick spectra dressed by Gaussians of line-widths, $\sigma.$ Specifically, we treat Chl *a*’s and Chl *b*’s as two level systems, assigning standard excitation energies ($14{,}900\,\mathrm{cm}^{-1}$ and $15,500\,\mathrm{cm}^{-1},$ respectively) and transition dipole moments [$4.5\,D$ and $3.4\,D,$ respectively (Knox and Spring 2003)]. We treat all four Cars as identical effectively three level systems: $S_{1}$ is placed in resonance with the $Q_{y}$ band of Chl *a*, while the energy of $S_{2}$ is adjusted to $20{,}100\,\mathrm{cm}^{-1}$ by subsequent fitting to an experimental spectrum. We set the $S_{2}$ dipole moment for all four Cars to that of Lut [i.e., $18\,D$ (Polívka and Sundström 2004)], while the $S_{1}$ dipole moment is set to $0.1\,D$ to represent the minimal yet non-negligible (and in some calculations rather typical Balevičius Jr. et al. (2017); Andreussi et al. (2015)) value. Additionally, $S_{1}$ and $S_{2}$ are each assigned manifolds of vibrational states representing the optically-coupled C-C and C=C vibrational modes (Balevičius Jr. et al. 2015). Without loss of generality we consider 3 levels each of the C–C and C=C modes (with frequencies $1100\,\mathrm{cm}^{-1}$ and $1500\,\mathrm{cm}^{-1},$ respectively) and their combinations. This effectively turns $S_{1}$and $S_{2}$ into two manifolds of 9 vibronic states. The dipole moments of these vibronic states are determined by weighting the corresponding purely electronic dipole moments by the Franck–Condon factors (we use identical displacements of 0.82 for each mode) (Bašinskaite et al. 2014; Balevičius Jr. et al. 2015). The excitonic effects arise from coupling two $S_{1}$ state manifolds to two excited states of Chl *a*’s [conf., (Bašinskaite et al. 2014)]. The energy redistribution is calculated by diagonalizing the total Hamiltonian, while the redistribution of the dipole moments is calculated using the corresponding diagonalizing matrix (Bašinskaite et al. 2014). Additionally, the line-widths $\sigma$ are mixed similarly to Eq. 3, akin to the excitonic mixing of transition energy correlation functions in the more rigorous line-shape function approach to computing linear absorption profiles (Renger and Marcus 2002).

## Results

### Energy relaxation and quenching within a Chl–Car pair and LHCII monomer: the FRET regime

We first consider the energy relaxation dynamics in the FRET regime, which was assumed in previous models of excitation quenching in light-harvesting proteins (Fox et al. 2017; Chmeliov et al. 2015; Ruban et al. 2007), as a bench mark for the HEOM calculations. Here we use the full spectral density of Lut evaluated in earlier work (Fox et al. 2017). Both in this and the following Sections we study three levels of energy relaxation. We first consider thermalization in an isolated dimer disregarding the energy escaping into the surroundings due to finite excited state lifetimes. Next, we include the intrinsic lifetimes in the description and evaluate the net excitation lifetime of the coupled pair. Lastly, we study the excitation lifetime of the LHCII monomer, considering two such dimers embedded within the complex.

**Fig. 3:**
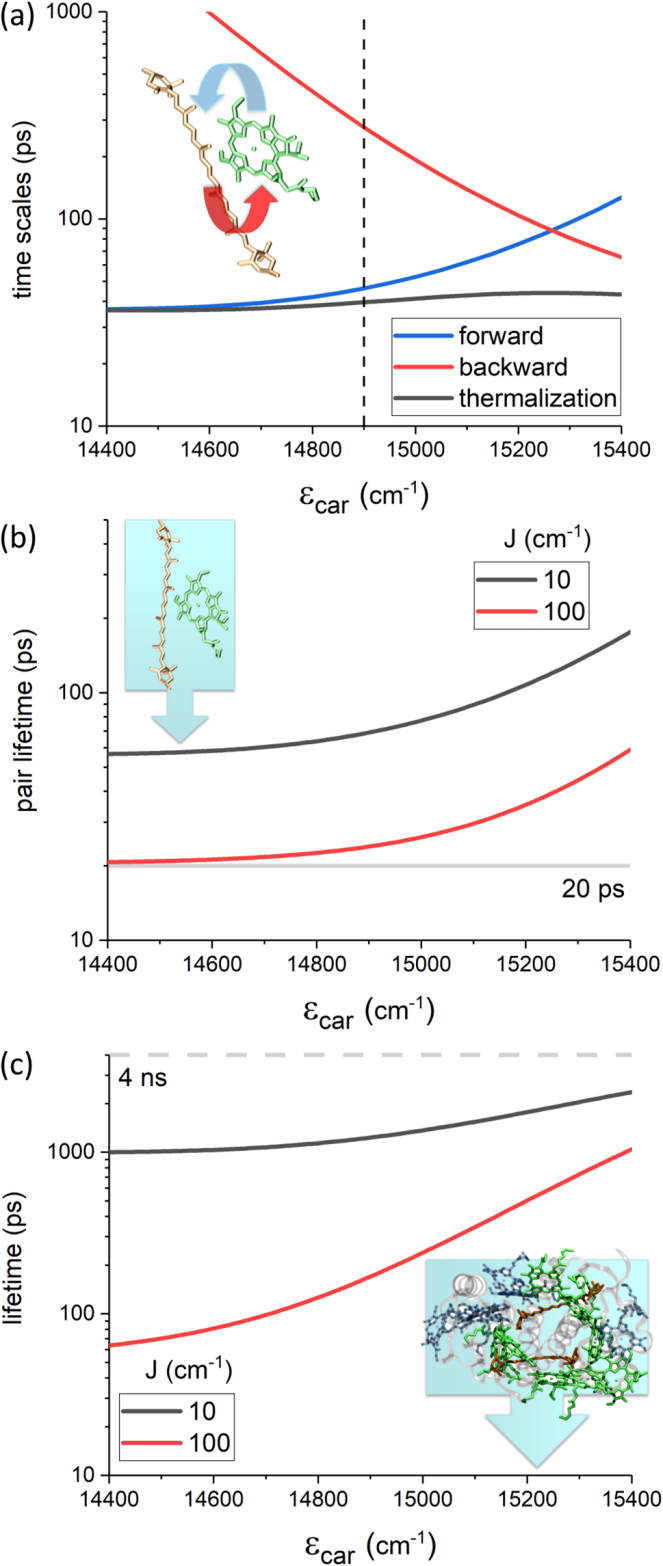
**a** The rates of forward-transfer (Chl *a*$\rightarrow$Lut, indicated by the blue arrow), back-transfer (Lut$\rightarrow$Chl *a*, red arrow) and thermalization within a Chl *a*-Lut pair, in the FRET regime, as a function of $\Updelta \varepsilon$ assuming $J=10\,\mathrm{cm}^{-1}.$ The behavior for larger couplings is identical but for a change in scale. We see that the overall thermalization time is largely insensitive to $\Updelta \varepsilon.$ The vertical dashed line represents the resonance point $\varepsilon_{\rm car}=\varepsilon_{chl}=14{,}900\,\mathrm{cm}^{-1}.$**b** Accounting for the intrinsic lifetimes of the $Q_{y}$ and $S_{1}$ allows for calculation of the overall excitation lifetime of the pigment pair (see inset, blue arrow implies excitation loss from the system) in the strongly (red, $J=100\,\mathrm{cm}^{-1}$) and weakly (black, $J=10\,\mathrm{cm}^{-1})$ coupled regimes. The minimum possible lifetime of $20\,\mathrm{ps},$ the intrinsic lifetime of $S_{1},$ is achieved for strong coupling and $\varepsilon_{\rm car}<<\varepsilon_{chl}.$**c** The lifetime of an LHCII monomer in which two Chl *a*–Lut quenching pairs are embedded (see inset). The gray dashed line represents the lifetime of detergent-solubilized LHCII ($4\,\mathrm{ns}$). Since this is also the lifetime of free Chl *a* in a similar solvent, $4\,\mathrm{ns}$ represents the lifetime of a totally unquenched system. We see that even small couplings result in a profound quenching even if $\varepsilon_{\rm car}>\varepsilon_{chl}$

The forward and backward excitation hopping rates and the overall thermalization rate are obtained directly from FRET. The dependencies of these three time scales on $\varepsilon_{\rm car}$ are shown in Fig. 3a. Only the case of $J=10\,\mathrm{cm}^{-1}$ is shown, because the FRET rates are proportional to $J^{2},$ hence, in the case of $J=100\,\mathrm{cm}^{-1}$ all the time scales are trivially faster by a factor of 100. The forward and backward hopping times are shown by blue and red lines in Fig. 3a, accordingly (the inset scheme shows the corresponding energy transfer directions). The vertical dashed line indicates the energy of Chl *a*, $\varepsilon_{\mathrm{chl}}=14{,}900\,\mathrm{cm}^{-1}.$ Notably, if $\varepsilon_{\rm car}<\varepsilon_{chl}$ the back-transfer slows down exponentially while, when $\varepsilon_{\rm car}>\varepsilon_{chl}$ forward transfer slows down and back-transfer is favored. We note that the forward and backward hopping times do not coincide when the pair are resonant ($\Updelta \epsilon =0$) due to the very large reorganization energy associated with the Car $S_{1}$ state (see supplementary material and Fox et al. (2017)). The vertical energy of $S_{1}$ must lie significantly above $Q_{y}$ ($\Updelta \varepsilon \sim +400\,\mathrm{cm}^{-1}$) for back-transfer to outpace forward transfer. Lastly, we see that while the ratio of the forward/backward rates (which determines the equilibrium populations of the two pigments) is strongly dependent on $\Updelta \epsilon,$ the time scale within which the equilibrium is established (the net thermalization time) is more-or-less flat across the probed energy range.

Next, we consider the Lut–Chl *a* dimer, taking into account the intrinsic excited state decay lifetimes of the two molecules (represented in the inset of Fig. 3b). The dependence of the dimer excitation lifetime, as defined in Eq. 6, on $\varepsilon_{\rm car}$ is shown in Fig. 3b for small and large *J*. Most notably the lifetime is vastly shorter than the 4 ns lifetime of the Chl *a* itself, regardless of the energetic position of $S_{1}$ with respect to $Q_{y}.$ Above all this demonstrates how any contact with a short-lived species (even in the complete absence of excitonic mixing) can reduce the overall lifetime, the short-lived state acting as an energy sink even if placed above the initial donor. For $\varepsilon_{\rm car}>\varepsilon_{chl}$ the pair lifetime increases because of the diminishing forward transfer to and increasing back-transfer from a high-lying $S_{1}$ (conf., Fig. 3a). For $\varepsilon_{\rm car}<\varepsilon_{chl}$ the pair lifetime approaches the theoretical limit of either the forward transfer time, $k_{chl\rightarrow car}^{-1},$ or the lifetime of the quencher (i.e., $\kappa_{\rm car}^{-1}=20\,\mathrm{ps}~$ here), or their mixture if the values are similar. Clearly, in the strong coupling case (red line) the quencher lifetime is the limiting factor. Such dependency indicates very efficient — rapid and irreversible — transfer to the quencher.

Lastly, we inspect the lifetime of an LHCII monomer. The excitation lifetime is again defined by Eq. 6, except this time the kernel of the Pauli master equation is given by Eq. 8. The dependence of the lifetime of the entire complex on $\varepsilon_{\rm car}$ is shown in Fig. 3c. The dashed line at the top of the graph indicates the 4 ns lifetime of the complex in the absence of any quenchers and corresponds to the lifetimes of free Chl *a*. Evidently, the existence of two quenching pairs internally coupled by as little as $10\,\mathrm{cm}^{-1}$ is capable of dropping the lifetime to 1–2 ns even for the highly unlikely scenario that $S_{1}$lies significantly above $Q_{y}.$ Notably, now strong coupling enables lifetime differences spanning an order of magnitude. Obviously there is no qualitative change in behavior as one approaches $\Updelta \varepsilon =0$ as FRET excludes a priori any coherent effects.

### Incorporating coherent effects

Now we will consider the ’true’ system dynamics by explicitly incorporating excitonic mixing effects, the degree of which will depend on $2J/\Updelta \varepsilon.$ We will investigate the dynamics around the resonance point $\Updelta \varepsilon =0,$ where $\Updelta \varepsilon$ is now defined between Chl *a *$Q_{y}$ and any vibronic level on Lut $S_{1}.$ The results for the three energy transfer situations are given analogously to the previous Subsection. The FRET results were updated using the OBO spectral density for the $S_{1}$ state in order to make them directly comparable to the HEOM results, although within this energy window they are qualitatively identical to the ones obtained with the full Car spectral density above. In the case of the FRET framework, all results are obtained (by definition) in the site basis, whereas in the HEOM case the results were calculated in the exciton basis (for procedural reasons), no matter how small the actual mixing.

**Fig. 4:**
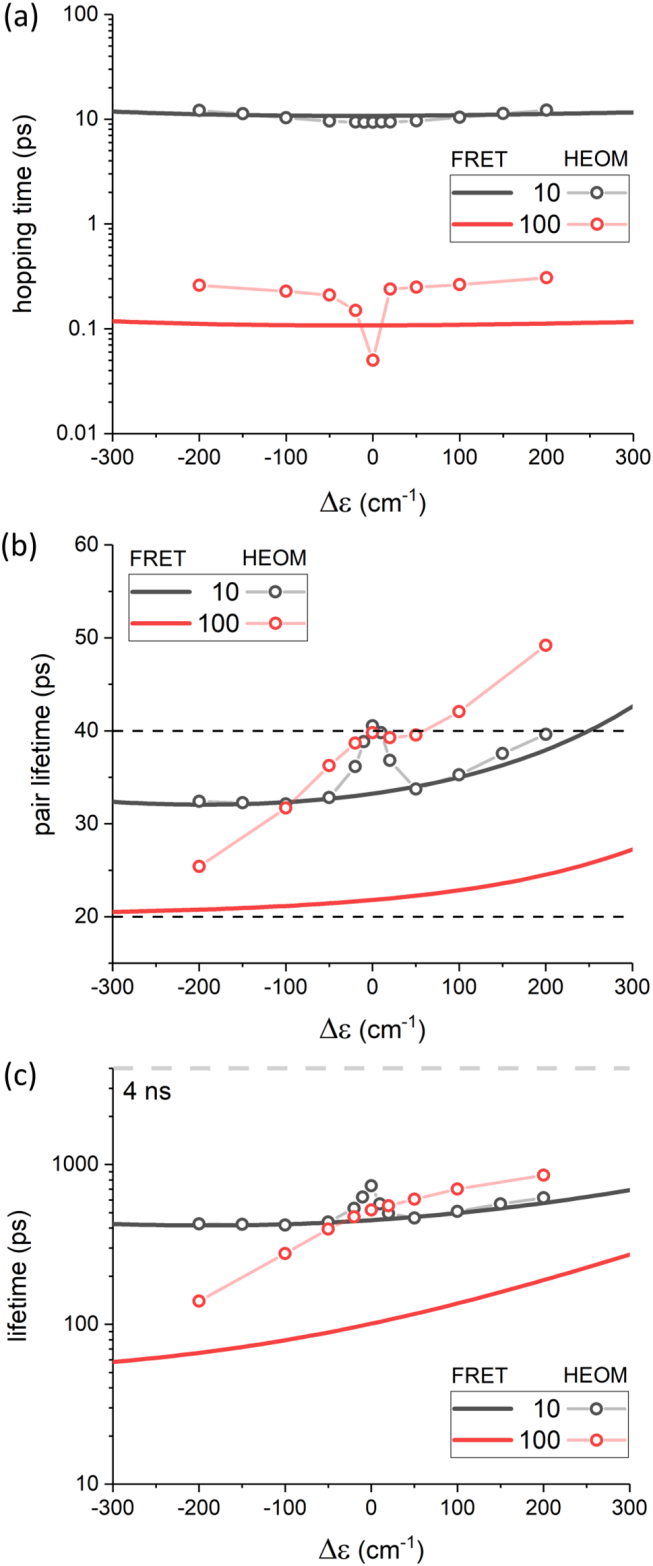
**a** Dependence of the thermalization time for the Chl *a*–Lut pair on $\Updelta \varepsilon$ for the strong (red, $J=100\,\mathrm{cm}^{-1}$) and weak (black, $J=10\,\mathrm{cm}^{-1}$) coupling regimes. We compare both the FRET regime (which excludes coherent effects) and the formally exact HEOM regime. A weakly-coupled dimer is clearly well described by FRET. Resonance effects become apparent for a strongly-coupled dimer and we see that out of resonance FRET under-estimates the thermalization time. **b** The excitation lifetime of a Chl *a*–Lut pair. The lower dashed line represents the intrinsic lifetime of Lut $S_{1}$ ($20\,\mathrm{ps}$), while the upper represents twice this value (the minimum possible quenching for a perfectly resonant excitonic quencher). For a weakly-coupled quencher FRET and HEOM give qualitatively identical result apart from the resonance point. The behavior of a strongly-coupled pair is more complex. For $\varepsilon_{\rm car}>\varepsilon_{chl}$ increasing coupling actually *decreases* the level of quenching. For $\varepsilon_{\rm car}<<\varepsilon_{chl}$ the FRET regime begins to be recovered. **c** Excitation lifetime for the LHCII monomer. The gray dashed line represents the lifetime of the system in the complete absence of quenching ($J=0$). The behavior is the same as for an isolated Chl *a*–Lut pair but the resonance features are less pronounced

The dependence of the thermalization time on the $$\Updelta \varepsilon$$ in Fig. 4a shows that for weak coupling FRET and HEOM give nearly identical results, which has already been reported (Balevičius Jr. et al. 2013). In the case of strong coupling the results are qualitatively identical outside the vicinity of the resonance, although FRET clearly overestimates the true (HEOM) thermalization rates outside of the resonance window. However, around $$\Updelta \varepsilon =0$$ a sharp deviation from the incoherent FRET scheme is observable and it deserves a word of caution. Particularly at $$\Updelta \varepsilon =0$$ the dynamics of the pair become highly non-trivial and the idea of a single transfer rate constant is not applicable in the usual sense. This is because at the degeneracy point the initial populations are equal, 0.5/0.5, for both the upper and lower excitonic states which differ in energy only by the Davydov splitting of 2*J*. An ultrafast thermalization over the Davydov splitting can be observed along with some further slower dynamics due to complex system–bath interactions and reorganization. We have included the time scale of these slower dynamics (obtained from exponential fitting of the population evolution) for the sake of consistency. Now that the hopping times are calculated using HEOM we can include the effect of any excitonic mixing of intrinsic decay rates, Eq. 3, in the our calculation of $$\tau_{\rm exc}.$$ The initial populations in the HEOM case are taken as $$\cos ^{2}\theta$$ for the Chl-like excitonic state and $$\sin ^{2}\theta$$ for the Car-like state, which is based on the excitonic mixing of dipole moments, which in turn redistributes the initial excitation (van Amerogen et al. 2000). The results are shown in Fig. 4b. The lower dashed line corresponds to the lifetime of the uncoupled Lut $$S_{1}$$ state, $$\tau_{\rm car},$$ and the upper dashed line corresponds to $$2\tau_{\rm car}.$$ Again, the case of a small resonance interaction gives identical results outside the resonance window of roughly 2*J*. Within the resonance window the lifetime approaches the theoretical limit of $$\tau_{\rm exc}\approx 2\tau_{\mathrm{car}}$$ (since $$\tau_{\mathrm{chl}}\gg \tau_{\mathrm{car}}$$), which can intuitively be understood by realizing that the excitation is perfectly delocalized across the two pigments, hence it resides on Lut only half of the time. As the $$S_{1}$$ increases in energy with respect to $$Q_{y},$$$$\tau_{\rm exc}$$ increases because of increasing back-transfer (as captured earlier by FRET). Although we use the term ’resonance window’ it is important to remember that there is no sharp boundary separating the nominally ’coherent’ and ’incoherent’ behaviors. In the case of large *J*, the HEOM and FRET results differ significantly within the considered energy window. Overall, due to a slower thermalization time, as seen in the upper panel, the excitation lifetime is longer than predicted by FRET. At perfect resonance the pair lifetime approaches the same theoretical limit of $$\approx 2\tau_{\mathrm{car}}$$as this is independent of *J*. Outside of the resonance window the dependence of $$\tau_{\rm exc}$$ on the $$\Updelta \varepsilon$$ is much steeper than for the weakly-coupled case. Interestingly, for $$\Updelta \epsilon >0$$ (i.e., $$\varepsilon_{\rm car}>\varepsilon_{chl}$$) the excitation lifetime of the pair is actually *longer* for small *J* than it is for large. This is due to larger resonance interactions inducing fast back-transfer. For $$\Updelta \epsilon <0$$ forward transfer becomes favorable, but quenching only significantly outpaces that seen for a weakly-coupled pair if $$\Updelta \epsilon<<J$$ (i.e.when excitonic effects become insignificant). Although we did not probed beyond $$\Updelta \epsilon \pm 200\,\mathrm{cm}^{-1}$$with HEOM the pair lifetime appears to approach the FRET value only when $$S_{1}$$ lies significantly lower than $$Q_{y}.$$

Lastly, we calculate $\tau_{\rm exc}$ for the LHCII monomer. Qualitatively, the results in Fig. 4c can be interpreted using all of the trends observed in the Chl *a*-Lut dimer. Quantitatively, however, several counter-intuitive effects are present. It appears, that in the case of small *J*, $\tau_{\rm exc}$ is *longest* at the resonance point (although it will clearly become longer in the unlikely case of $\Updelta \epsilon >300\,\mathrm{cm}^{-1}$). This is completely contrary to the idea of excitonic interactions being an essential feature of the quenching mechanism. Additionally, we see that increasing the coupling by an order of magnitude ($J=10\rightarrow 100\,\mathrm{cm}^{-1}$) does not generally result in a qualitative increase in quenching. the only exception is when $\Updelta \epsilon<<-J,$ where the incoherent limit applies and the lifetime approaches that predicted by FRET (solid red line in Fig. 4). Regardless of the magnitude of *J* the overall quenching behavior is essentially determined by the rate of back-transfer from the quencher and the excitonic mixing of lifetimes barely plays any role.

### The effect of excitonic mixing on the absorption spectrum of LHCII

**Fig. 5:**
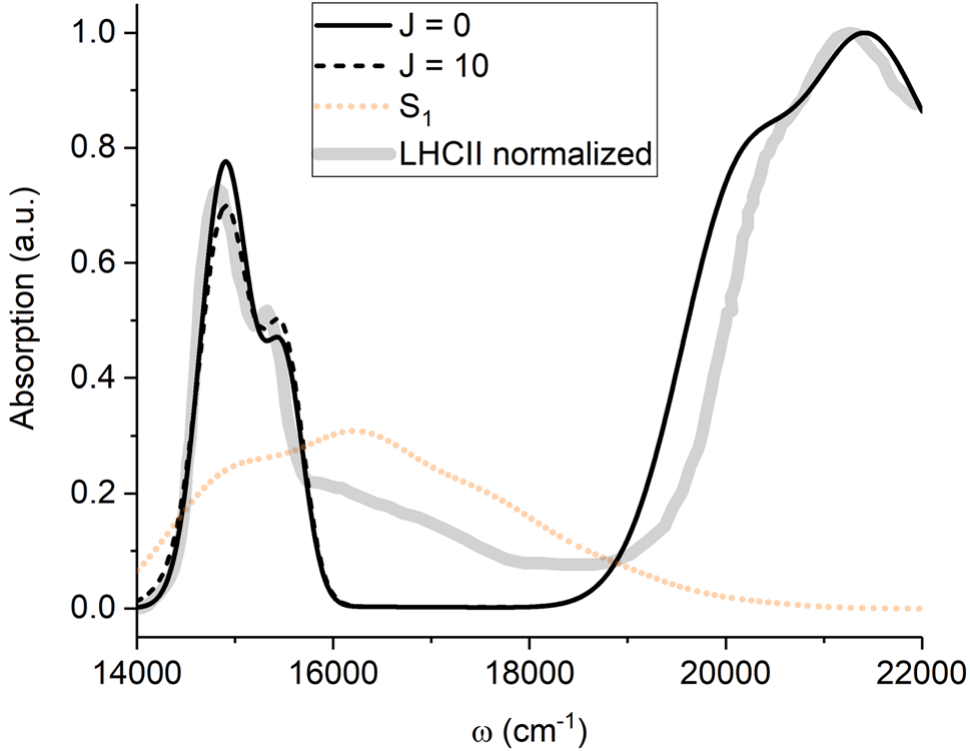
Absorption spectrum of the LHCII monomeric sub-unit. The experimental absorption spectrum [taken from (Croce et al. 1999)] is shown as a faint gray line. The black line shows the calculated spectrum in which we have assumed no Chl–Car coupling. The dashed line indicates the calculated spectrum in which both central Chl–Lut pairs are at resonance ($\Updelta \varepsilon$=0) and weakly-coupled ($J=10\,\mathrm{cm}^{-1}$). We note that it is not our intention to reproduce the complete experimental absorption spectrum, hence the vibronic/$Q_{x}$ features of the Chls ($16{,}000\,\mathrm{cm}^{-1}<\omega <18{,}000\,\mathrm{cm}^{-1}$) are missing. Similarly the Car $S_{2}$ absorption is only schematic and not carefully fitted. For clarity the Car $S_{1}$ line-shape is shown with a very exaggerated amplitude and is in reality undetectable. The calculated spectrum for $J=100\,\mathrm{cm}^{-1}$ is not shown, as it is essentially identical to that of $J=10\,\mathrm{cm}^{-1}$

We finally calculated the absorption spectrum of monomeric LHCII with and without two Chl *a*-Lut quenchig pairs. The resulting spectra are shown in Fig. 5. The full black line corresponds to the sum of 8 non-interacting Chl *a*’s, 6 Chl *b*’s and 4 “generalized” Cars. The only fit parameter (apart from displacements/Franck–Condon factors for Car states) was the widths of the Gaussians, which were determined to be $$250\,\mathrm{cm}^{-1},$$$$200\,\mathrm{cm}^{-1}$$ and $$590\,\mathrm{cm}^{-1}$$ for Chl *a*, Chl *b* and the Car, respectively. The experimental spectrum used for fitting and reference is taken from (Croce et al. 1999) and shown as a thick gray line. It is noteworthy that the region of $$16{,}000{-}18{,}000\,\mathrm{cm}^{-1}$$ is clearly underfitted due to the neglect of the Chl $$Q_{x}$$ states and/or the vibronic features of $$Q_{y}.$$ Also, the $$S_{2}$$ region around $$20{,}000\,\mathrm{cm}^{-1}$$ shows only a rough fit, which is mostly the result of neglecting differences between different Car species present in LHCII. Neither of these regions is pertinent to this work. The $$Q_{y}$$/$$S_{1}$$ region of the spectrum is surprisingly well-described given the simple nature of the model (we neglect any state mixing or transition dipole redistribution amongst the Chls. We show the position and line-shape of $$S_{1}$$ with the orange line although to make it visible in the figure we have used a massively exaggerated $$S_{1}$$ transition dipole moment of $$10\,D.$$ In reality the $$S_{1}$$signal is essentially absent in the overall spectrum despite the non-negligible dipole strength of $$0.1\,D$$ (since absorption scales as the square of the dipole moment). The black dashed line shows the effect of introducing a small coupling ($$J=10\,\mathrm{cm}^{-1}$$) in each of the two Chl *a*–Lut pairs where we have assumed both are at perfect resonance ($$\Updelta \varepsilon$$=0). The Chl *a *peak shifts ($$\pm J$$) are essentially undetectable against the background of the non-coupled Chl’s and remain so if $$J=100\,\mathrm{cm}^{-1}$$(not shown). There is some small effect from the redistribution of oscillator strength which is independent of the scale of *J* (since the quenchers are assumed to be at maximal resonance anyway). Naively, there appears to be some exchange in relative peak intensity of Chl *a* ($$14{,}900\,\mathrm{cm}^{-1}$$) and Chl *b* (the shoulder at $$15{,}500\,\mathrm{cm}^{-1}$$). However, this is due to the fact that mixing between $$Q_{y}$$ and $$S_{1}$$ results in broadening of the absorption line-shape of the two Chl *a* in the quenching pairs. The fact that this manifests as changes around the Chl *b* peak is coincidental.

## Discussion

There have been previous theoretical studies of the influence of excitonic effects on the dynamics of excitation quenching in a general molecular heterodimer (Balevičius Jr. et al. 2012) and later in phthalocyanine–carotene dyads (Balevičius Jr. et al. 2013). Here, however, we are interested in the relevance of excitonic effects to excitation quenching in the Chl *a*–Lut domains of LHCII as it pertains to the NPQ mechanism. Our previous model of quenching in the LHCII crystal structure was purely incoherent because of the model assumptions, albeit seemingly reasonable ones. The excitonic model was initially a hypothesized to explain the presence of short lifetime components in the fluorescence kinetics of of LHCII aggregates (van Amerongen and van Grondelle 2001). It later seemed to explain a number of spectroscopic observations (Bode et al. 2009; Liao et al. 2010a, b). However, clearly neither is a complete description of the real dynamics which have been observed to have characteristics of both (Holleboom and Walla 2014). Clearly a more general theoretical approach was needed.

### Quantitative and qualitative differences between coherent and incoherent quenching

Throughout $\Updelta \varepsilon$ was treated as a free parameter while we chose $J=10\,\mathrm{cm}^{-1}$ and $100\,\mathrm{cm}^{-1}$ as representative of weakly- and strongly-coupled regimes between which it is trivial to interpolate. Obviously, excitonic mixing is possible for either but the strongly-coupled case gives us a larger ‘resonance window’, $\Updelta \varepsilon \approx \pm J,$ in which to study it. The remaining parameters, those associated with the system–bath interaction, are set to the values that (to date) given the most accurate description of Chl and Car spectral properties. When considering quenching in LHCII we assume the dynamics in the larger Chl pool are as described previously (Chmeliov et al. 2015). We note that the Chl dynamics within LHCII have been modeled with higher precision, e.g., employing HEOM (Kreisbeck et al. 2014; Wu et al. 2015), but these finding are not critical to our current discussion which focuses on the effect of connecting Lut $S_{1}$state to this Chl pool. For the moment we consider these effects generally. First of all, by comparing the thermalization times given by FRET theory and HEOM (see Fig.4a), we confirm earlier observations that, for a weakly-coupled system, the simple FRET approach fully reproduces the exact dynamics given by HEOM, even capturing the effects of a relatively long bath memory (i.e., a non-Markovian system) (Balevičius Jr. et al. 2013). For a strongly-coupled pair FRET fails to quantitatively reproduce the dynamics. It clearly under-estimates the thermalization time, even when excitonic effects should be minimal ($|\Updelta \varepsilon |>J$), although it captures the lack of dependence on $\Updelta \varepsilon$ in this region. HEOM shows a sharp decrease in thermalization time around $\Updelta \varepsilon =0,$ this is due to the presence of excitonic effects that are, naturally, not captured by FRET. Importantly, we see that the thermalization time is far more sensitive to *J* than to $\Updelta \varepsilon.$ This is a direct result of the very large reorganization energy of the Car $S_{1}$ state which supresses back-transfer of energy to $Q_{y}.$

For all values of *J* and $\Updelta \varepsilon$ probed, in both the dimer and LHCII, non-zero Chl-Car coupling results in profound quenching (see Fig. 4b, c). About $\Updelta \varepsilon =0$ the HEOM calculations explicitly demonstrate quenching as a product of lifetime mixing. For the dimer the lifetime is $\sim 2\tau_{\mathrm{car}}$ (since $\tau_{\rm Car}<<\tau_{\rm Chl}$) as analytically demonstrated earlier (Balevičius Jr. et al. 2013). This is the case in both the weakly- and strongly-coupled cases, although the behavior around the resonance point is different. For the weakly-coupled case the effect of lifetime mixing clearly emerges in the immediate vicinity of $\Updelta \varepsilon =0.$ However, outside this narrow window the state mixing becomes negligible and we can attribute the quenching solely to $S_{1}.$ The small resonance coupling and the large reorganization energy of $S_{1}$strongly suppress back-transfer, meaning energy transfer essentially occurs unidirectionally to $S_{1}.$ Consequently, the pair lifetime falls below the $\sim 2\tau_{\mathrm{car}}$ excitonic limit even, rather counter-intuitively, when$S_{1}$ lies above $Q_{y}.$ The mixing effect is also clearly observable for the LHCII monomer. As expected, we see that FRET theory gives an entirely sufficient description of the dynamics of both the dimer and LHCII outside the resonance window. The strongly-coupled case is more complicated. At the resonance point $\tau_{\rm exc}\sim 2\tau_{\rm Car}$ as in the weakly-coupled case. The efficiency of quenching under conditions of perfect state mixing is independent of the magnitude of *J* (providing $J>0$). Around the resonance point the dependence of lifetime on $\Updelta \varepsilon$ reveals no sharp transition between excitonic and incoherent quenching. This is due to the fact that the strong coupling means that the rate of back-transfer becomes comparable to $\tau_{\rm exc},$ which smoothes the dependence of $\tau_{\rm exc}$ on $\Updelta \varepsilon.$ As with the weakly-coupled case there is still significant quenching in the absence of excitonic effect, even when $S_{1}$ lies above $Q_{y}.$ The most surprising result is that increasing the coupling by an order of magnitude does not result in a concomitant increase in quenching, as one would predict from FRET. The only exception would be if $S_{1}$ lay significantly below $Q_{y}$ where the trends in Fig. 4b, c show the true (HEOM) dynamics converging on those predicted by FRET. Dynamical details aside significant quenching occurs regardless of where the system sits between the coherent and incoherent limits.

### Contribution of excitonic quenching to the NPQ mechanism

Important points to consider when discussing excitonic quenching are: Does it represent a uniquely effective channel for excitation dissipation? Do the expected parameters in the real system, in this case LHCII, allow for significant lifetime mixing? If it does occur are the excitonic peak shifts and mixing of oscillator strengths small enough to have gone undetected in the linear absorption profile of LHCII? Despite the generality of our model it yields strong constraints on the expected behavior of the NPQ quencher. It is apparent that neither excitonic mixing nor energy transfer represents unique or necessary pathways for quenching and there appears to be no phenomenological difference between the two. Moreover, it appears it is essentially impossible for the system to switch between quenched and unquenched states by tuning the energy gap about $\Updelta \varepsilon =0.$ In fact even very large changes in energy gap do not produce any drastic reduction in quenching. It is clear then that quenching is determined almost entirely by *J*.

The actual values of $$\Updelta \varepsilon$$ and *J* that apply to LHCII are difficult to obtain. While $$\varepsilon_{chl}$$ for Chl* a* in LHCII is easily measured at $$\sim$$ 14,900 $$\mathrm{cm}^{-1},$$$$\varepsilon_{car}$$ for Lut is less well-defined due to $$S_{1}$$ being optically-forbidden. Two-photon absorption on Lut in native LHCII gives a value $$\sim$$ 15,300 $$\mathrm{cm}^{-1}$$ although this is taken from fitting a single line to the sharpest peak in the data (a higher energy vibronic peak) (Walla et al. 2000). Fitting the cleaner two-photon spectrum of Lut in octanol (Walla et al. 2002) we previously obtained a value of $$\sim$$ 14,000 $$\mathrm{cm}^{-1}$$ (with a second vibronic peak at $$\sim$$ 15,300 $$\mathrm{cm}^{-1}$$) (Fox et al. 2018) which matches values obtained from $$S_{1}$$ excited state absorption (Polívka and Sundström 2004). Although the precise value is not well defined it is reasonable to assume that Chl *a*$$Q_{y}$$ lies somewhere between the $$S_{1}$$ 0-0 and first vibronic transitions. Some degree of excitonic mixing will obviously be present, although in terms of NPQ, the absolute value makes little difference.

With regard to *J* our earlier semi-empirical models of LHCII estimated this to be $10{-}20\,\mathrm{cm}^{-1}$ (Chmeliov et al. 2015; Fox et al. 2017) and a more recent, high-level, calculation has placed it at $21.9\,\mathrm{cm}^{-1}$ (Khokhlov and Belov 2019). One problem is that $S_{1}$ is rigorously optically-forbidden, which means that these calculations cannot be corrected against spectroscopic data and are therefore open to the intrinsic numerical errors of quantum chemistry. However, $S_{1}$ is not merely dipole-forbidden (like the higher energy cis-band states) but lacks a significant one-electron transition density (it is predominantly a two-electron transition). This means, even at very close distances and even taking into account the short-range exchange and overlap interactions, the coupling of $S_{1}$ to other transitions is significantly limited Wei et al. (2019). $J=100\,\mathrm{cm}^{-1}$ is typical of the strongest Chl-Chl couplings within LHCII Renger and Müh (2013) and it seems unlikely that $S_{1}-Q_{y}$ coupling will reach this level. However, given favorable orientations it could be possible for the coupling to exceed the $21.9\,\mathrm{cm}^{-1}$ lower limit. The magnitude of the coupling determines two things, the rate of thermalization within the pigment pair and the size of the resonance window about $\Updelta \varepsilon =0.$ Above a threshold of $J\sim 10\,\mathrm{cm}^{-1},$ the magnitude of *J* does not strongly-determine the overall efficiency of quenching. This is because at resonance the lifetime is independent of *J* and increasing *J* increases the resonance window. This actually offers a possible explanation for an apparent inconsistency. The recently observed ultrafast ($<0.4\,\mathrm{ps}$) Chl *a*$\rightarrow$ Lut hopping time in quenched LHCII (Son et al. 2019) suggests a coupling rather larger than $J=10\,\mathrm{cm}^{-1}.$ The FRET framework would suggest that this is at odds with the modest $(\tau_{\rm exc}=0.4{-}1.0\,\mathrm{ns}$) level of quenching observed in isolated LHCII trimers. However, HEOM shows that strong coupling/fast hopping does not translate into strong quenching.

In this work we treated *J* and $\Updelta \varepsilon$ as parameters with fixed values although in the real system they will naturally fluctuate. Fast ($t<1/k_{ab}$) fluctuations in $\varepsilon_{\rm car}$ and $\varepsilon_{\rm chl}$ (and therefore $\Updelta \varepsilon$) due to the system-bath interaction actually drive excitation relaxation and enter our HEOM calculations implicitly via the spectral density. However, they are too fast to be resolved by the quenching mechanism itself. At the other end of the scale we have the $1{-}10\,\mathrm{s}$ fluorescence intermittency seen in single-molecule experiments. These are related to switching between conformational states and probably reflect the NPQ switch itself (Chmeliov et al. 2013). We have shown here that these must be due to ’on/off’ switching of *J* rather than any tuning of $\Updelta \varepsilon.$ The most interesting fluctuations are those that occur within the quenched conformation on a time scale $t\ge \tau_{\rm exc}.$ These are due to static disorder and are observed as the fast modulations in fluorescence intensity within the meta-stable ’dark’ states in single-molecule experiments. Physically they originate from changes to the pigment binding pockets witch result in distortions to pigments and changes in their relative orientation. A single LHCII trimer will therefore randomly sample the parameter space that we have here explored systematically. In the ensemble we can expect some statistical mixture and indeed time-resolved measurements have shown that ’the’ conformation of LHCII is composed of several distinct sub-populations(Schlau-Cohen et al. 2015).

## Conclusions

The discussion of excitonic Chl–Car interactions and Chl $\rightarrow$ Car energy transfer as distinct mechanism in NPQ is somewhat redundant. Neither represent uniquely effective pathways for excitation quenching and both are merely approximate representations of the real dynamics in different hypothetical limits. The real dynamics exist in an intermediate regime but we have shown the degree to which excitonic effect are or are not present has almost not functional consequences for quenching. We do show that in LHCII (as in dyadic systems) quenching cannot be switched between light-harvesting and photoprotective states by fine tuning of the Chl $Q_{y}-$Car $S_{1}$ energy gap about the resonance point. The Car can induce significant quenching even if the $S_{1}$state lies above the donor state. The definitive parameter in NPQ is the Chl–Car coupling, *J*, which needs to be very small if quenching is to be abolished and the light-harvesting state recovered. Due to the intrinsically quenched nature of Cars only a small coupling $J\sim 10\,\mathrm{cm}^{-1}$ is needed to produce the observed levels of quenching. 2D spectroscopy seems to suggest that the coupling is somewhat stronger than this but, rather counter-intuitively, this does not translate to stronger quenching, a phenomenon not captured by FRET. We do not suggest that excitonic effects are absent from NPQ and in fact there is compelling spectroscopic evidence that they are present to some degree. This work shows that the precise dynamics of quenching may vary significantly while its overall kinetics does not. This is in keeping with the idea the biological processes are not generally finely tuned and offers an explanation for previous observations of quenching with a mixed coherent/incoherent character (Holleboom and Walla 2014).

## Electronic supplementary material

Below is the link to the electronic supplementary material.

Supplementary material 1 (DOCX 130 KB)
