# Supplementary material for: Excitation quenching in chlorophyll–carotenoid antenna systems: ‘coherent’ or ‘incoherent’
Source: Photosynth Res. 2020 Apr 8;144(3):301–15. doi: 10.1007/s11120-020-00737-8 (PMC7239839; doi:10.1007/s11120-020-00737-8)
Supplement: Supplementary file 1 — Supplementary material 1 (DOCX 130 KB) [file 11120_2020_737_MOESM1_ESM.docx]

**Excitation quenching in chlorophyll–carotenoid antenna systems: `coherent' or `incoherent'**

Vytautas Balevičius Jr.^1^ and Christopher D. P. Duffy^1,#^

^1^School of Biological and Chemical Sciences, Queen Mary University of London, Mile End Road, London E1 4NS, UK

^#^c.duffy@qmul.ac.uk

**Supplementary Material**

***1. Details on the FRET approach to modelling excitation relaxation in the Chl a-Lut heterodimer***

FRET theory explicitly yields the rate constant for intermolecular excitation hopping (site basis). They are given by the Fermi Golden Rule,

$$k_{m\to n}=2\left| J_{mn} \right|^{2}\mathrm{Re}\int_{0}^{\infty} dt A_{n}\left( t \right)F_{m}^{*}\left( t \right) (1)$$

where $J_{mn}$ is the resonance coupling between nominal donor $m$ and acceptor $n$, while $A_{n}\left( t \right)$ and $F_{m}\left( t \right)$ are the acceptor absorption and donor fluorescence linear response functions respectively. The integral is essentially a density of states distribution and is entirely equivalent to the more familiar spectral overlap integral (in the frequency domain). Expressing this in the time domain has the benefit of the response functions having simple analytical forms,

$$A_{n}\left( t \right)=\exp\left( -i\frac{E_{n}}{\hbar}t-g_{n}(t) \right) (2)$$

$$F_{m}\left( t \right)=\exp\left( -i\frac{\left( E_{m}-2\lambda_{m} \right)}{\hbar}t-g_{m}^{*}(t) \right) (3)$$

Where $E_{m/n}$ is the site energy (local molecular transition energy) of the donor/acceptor, $\lambda_{m}$ is the reorganization energy of the acceptor ($2\lambda_{m}$ is the Stokes shift) and $g_{m/n}(t)$ are the associated line-broadening functions. The line-broadening function can be expressed generally as,

$$g_{i}\left( t \right)=\int_{0}^{\infty} \frac{d\omega}{\pi\omega^{2}}C_{i}^{''}\left( \omega\right)\left[ \left( 1-cos(\omega t) \right)\coth\left( \frac{\hbar\omega}{2k_{B}T} \right)+i\left( \sin\left( \omega t \right)-\omega t \right) \right] (4)$$

This function (and therefore the response functions and, ultimately, the spectral line shapes) depends on the spectral density, $C_{m/n}^{''}\left( \omega\right)$, of the chromophore. $C_{i}^{''}\left( \omega\right)$ characterizes the system-bath interaction and is essentially the distribution of the vibrational frequencies of the bath weighted by their couplings to the electronic degrees of freedom that constitute the system. In our case the system is the local electronic transition of the chromophores and the bath is composed of the nuclear vibrations of the chromophores and the surrounding medium. For the Chl *a* in our quenching dimer we use the very well established spectral density of Renger and Marcus^1^. This was obtained by fitting an ansatz function to hole-burning data. For the Lut we employ our previous spectral density function,

$$C_{Lut}^{''}\left( \omega\right)=\frac{2\lambda_{0}\gamma_{0}\omega}{\omega^{2}+{\gamma_{0}}^{2}}+\sum_{k=1,2} \frac{2\lambda_{k}\gamma_{i}{\omega_{k}}^{2}\omega}{\left( \omega^{2}-{\omega_{k}}^{2} \right)^{2}+{\gamma_{k}}^{2}\omega^{2}} (5)$$

This is composed of three ‘modes’. The two terms in the summation represent two under-damped high frequency vibrational modes. These represent the optically-coupled C=C and C-C stretching modes of the conjugated backbone. The first term is an over-damped mode (Drude model) which represents all other interactions with the bath. These modes are each associated with a reorganization energy, $\lambda$, and a correlation time, $\gamma^{-1}$, for fluctuations. The under-damped (oscillatory) modes are additionally associated with well-defined characteristic frequencies, $\omega_{k}$. These parameters are obtained by visually fitting the experimental absorptions spectrum, $A\left( \omega\right)$, which is simply the Fourier Transform of the response function, $A\left( t \right)$. For the Lut S_1_ state we fit the 2-photon absorption (2PA) profile lutein in octanol^2^. We show this in Supp. Fig. 1 with the more usual S_2_ (1PA) spectral fit for comparison. Although the 2PA data are sparse and noisy compared to the 1PA trace we see a similar spectral width and hints of a similar vibronic structure.

Although we can’t directly decompose $A\left( \omega\right)$ in terms of the parameters appearing in $C_{Lut}^{''}\left( \omega\right)$, the overall (very large) width of the spectrum is mostly attributed to the reorganization energies, which are necessarily themselves very large. The following parameters are taken directly from the fit: $\lambda_{0}=450 \mathrm{cm}^{-1}$, $\gamma_{0}=53 \mathrm{cm}^{-1}$ ($\sim100 \mathrm{fs}$) $\lambda_{1}=\lambda_{2}=900 \mathrm{cm}^{-1}$, and $\gamma_{1}=\gamma_{2}=300 \mathrm{cm}^{-1}$ ($\sim600 \mathrm{fs}$). To avoid over-fitting the values of $\omega_{1}=1530 \mathrm{cm}^{-1}$ and $\omega_{1}=1156 \mathrm{cm}^{-1}$ were taken from (ground state) resonance Raman spectroscopy.

***2. Details on the HEOM approach to modelling excitation relaxation in the Chl a-Lut heterodimer***

A general discussion of a molecular heterodimer can be found in ^4^ and references therein. A brief description of the basic model and assumptions follows.

Our Chl *a*-Lut quenching dimer is modelled as a coupled two-level system which is further coupled to the bath. These two levels (sites) are the electronic excited states of our two chromophores and the states $|\left. i \right\rangle=|\left. a \right\rangle$, $|\left. b \right\rangle$ constitute our site basis. The system (spin-boson) Hamiltonian is defined,

$$H_{S}=\sum_{i=a,b} \left( \varepsilon_{i}^{0}+\lambda_{i} \right)|\left. i \right\rangle\left\langle i \right.|+\sum_{i\neq j} J_{ij}|\left. i \right\rangle\left\langle j \right.| (6)$$

where $\varepsilon_{i}^{0}$ and $\lambda_{i}$ are the (relaxed) site energy and reorganization energy of the i^th^ site respectively. The quantity $\varepsilon_{i}=\varepsilon_{i}^{0}+\lambda_{i}$ is therefore the electronic transition energy and is equivalent to $E_{i}$ in the previously describe FRET analysis. Note that we do not explicitly include the electronic ground states of the two chromophores in the relevant system. Instead they enter implicitly as empirical excitation lifetimes (see below). The local nuclear degrees of freedom that constitute the bath are described by the Hamiltonian,

$$H_{B}=\sum_{i=a,b} \left( T_{i}\left( p \right)+V_{ig}\left( q \right) \right) (7)$$

where $T_{i}\left( p \right)$ and $V_{ig}\left( q \right)$ denote the kinetics energy and ground state potential energy surface of the i^th^ site respectively. $p$ and $q$ denote conjugate momenta and coordinate variables respectively. The system-bath Hamiltonian is defined,

$$H_{SB}=\sum_{i=a,b} \Delta V_{i}(q)|\left. i \right\rangle\left\langle i \right.| (8)$$

$\Delta V_{i}(q)$ is the *energy gap operator*,

$$\Delta V_{i}\left( q \right)=V_{ie}\left( q \right)-V_{ig}\left( q \right)-\lambda_{i} (9)$$

where $V_{ie}\left( q \right)$ is the excited state potential energy surface of the i^th^ site. $\Delta V_{i}\left( q \right)$ describes the thermal fluctuations of the gap between the ground and excited state potential energy surfaces which are characterized by a correlation time, ${\gamma_{i}}^{-1}$. As such, ${\gamma_{i}}^{-1}$ determines the timescale for the dissipation of the reorganization energy, $\lambda_{i}$, on the excited state of its respective chromophore. $\lambda_{i}$ has the statistical definition,

$$\lambda_{i}=\left\langle V_{ie}\left( q \right)-V_{ig}\left( q \right) \right\rangle_{q} (10)$$

where $\left\langle\ldots\right\rangle_{q}$ denotes an average over the equilibrium bath.

The dynamics of this open quantum system are described by the evolution of the Reduced Density Operator (RDO),

$$\rho\left( t \right)=\mathrm{Tr}_{q}W\left( t \right) (11)$$

where $W\left( t \right)$ is the full Density Operator of the system plus the bath and the trace is over the bath (nuclear) degrees of freedom. The evolution of $\rho\left( t \right)$ is given by the Liouville equation,

$$\frac{d}{dt}\rho\left( t \right)=-i\mathcal{L}\rho\left( t \right)\mathcal{+D}\rho\left( t \right)\mathcal{+K}\rho\left( t \right) (12)$$

The *Liouvillian* super-operator, $\mathcal{L}$, describes the deterministic (unitary) evolution of the RDO barring any interactions with the bath,

$$\mathcal{L}\rho\left( t \right) \equiv\frac{1}{\hbar}\left[ H_{S}, \rho\left( t \right) \right] (13)$$

where $\left[ A, B \right]=AB-BA$ denotes the commutator. Both $\mathcal{D}$ and $\mathcal{K}$ quantify the irreversible dynamics of the system which arise from interactions with the bath. $\mathcal{K}$ represents relaxation of the system to the ground state through processes such as fluorescence and interconversion. Since the ground state of the dimer is not explicitly included in the model, $\mathcal{K}$ is defined phenomenologically,

$$\mathcal{K}\rho\left( t \right)=-\sum_{i=a,b} \frac{{\tau_{i}}^{-1}}{2}\left\{ \left| \left. i \right\rangle\left\langle i \right. \right|,\rho\left( t \right) \right\} (14)$$

where $\tau_{i}$ are the intrinsic lifetimes of the individual chromophore excited states (described in the main article) and $\left\{ A,B \right\}=AB+BA$ denotes the anti-commutator. This ensures that the diagonal elements of the RDO, the classical populations, $P_{i}\left( t \right)=\rho_{ii}(t)$, decay with rate ${\tau_{i}}^{-1}$ while the off-diagonal elements, the coherences, $\rho_{ij}(t)$, decay with a rate $\left( k_{i}+k_{j} \right)/2$. $\mathcal{D}$ describes dissipation/reorganization and dephasing on the excited states. This the crucial term and the various approaches to modelling open quantum systems are largely defined by how they treat this highly non-trivial term. Here we use the HEOM approach which is described in more detail below. Assuming we have constructed our model we need only define our initial conditions and then propagate our system according to the equations of motion. We assume that,

$$W\left( 0 \right)=\rho\left( 0 \right)\otimes W_{eq} (15)$$

where,

$$W_{eq}=\frac{e^{-\beta H_{B}}}{\mathrm{Tr}_{q}e^{-\beta H_{B}}} (16)$$

is the density operator of the bath at thermal equilibrium ($\beta=1/{k_{B}T}$). This is essentially an assumption that there are no correlations between the system and bath prior to the initial optical excitation. We assume that the initial excitation occurs at $t=0$ due to interaction with an ultra-short optical pulse which excites only the optically-active Chl *a* transition. Our initial condition is therefore,

$$\rho_{ij}\left( 0 \right)=\delta_{ij}\delta_{i,Chl a} (17)$$

The HEOM approach treats the system-bath interactions (specifically those characterized by $\mathcal{D}$ in our model) non-perturbatively while assuming Gaussian bath fluctuations^5,6^. The effect of the bath on the system is totally determined by the *energy gap correlation function*,

$$C_{ij}\left( t \right)=\left\langle\Delta V_{i}(t)\Delta V_{j}(0) \right\rangle_{q} (18)$$

where,

$$\Delta V_{i}\left( t \right)=e^{iH_{B}t}\Delta V_{i}\left( q \right)e^{-iH_{B}t} (19)$$

is the energy gap operator transformed into the interaction picture. $C_{ij}\left( t \right)$ is related to the spectral density of the bath by the fluctuation-dissipation theorem,

$$C_{ij}\left( t \right)=-\frac{1}{\pi}\int_{-\infty}^{\infty} d\omega e^{-i\omega t}C_{ij}^{''}(\omega)\frac{1}{e^{-\beta\omega}-1} (20)$$

Here we assume that the bath fluctuations at one chromophore are uncorrelated with those at the other,

$$C_{ij}^{''}\left( \omega\right)=\delta_{ij}C_{i}^{''}\left( \omega\right) (21)$$

and $C_{i}^{''}\left( \omega\right)$ is that of an over-damped Brownian oscillator (ODO),

$$C_{i}^{''}\left( \omega\right)=\frac{2\lambda_{i}\gamma_{i}\omega}{\omega^{2}+{\gamma_{i}}^{2}} (22)$$

The central aim of HEOM is to approximate the integral in Eqn. (20) as an exponential series,

$$C_{i}\left( t \right)=\sum_{k=0}^{K} c_{ik}e^{-\gamma_{ik}t}+\delta C_{iK}\left( t \right) (23)$$

where $\delta C_{iK}\left( t \right)$ is the deviation of the approximate correlation function from the true one. The index $k=0$ denotes the pole of the ODO spectral density, $C_{ij}^{''}(\omega)$, while $k=1,\ldots,K$ indicate the poles of the Bose-Einstein distribution, $1/\left( e^{-\beta\omega}-1 \right)$. There are several schemes for choosing coefficients, $c_{ik}$, and exponents, $\gamma_{ik}$, but, as in previous work^4^ we adopt the approach reported by Hu et al.^7^ as it was shown to be optimal in the case of an ODO. The coefficients are split into real and imaginary parts, $c_{ik}=c_{ik}^{R}+ic_{ik}^{I}$, and are defined,

$$c_{ik}^{R}=\left\{ \begin{aligned} \frac{2\lambda_{i}}{\beta}+\sum_{k^{'}=1}^{K} \frac{4\eta_{k'}\lambda_{i}{\gamma_{i}}^{2}}{\beta\left( {\gamma_{i}}^{2}-{\varphi_{k'}}^{2} \right)}-2R_{K}\beta\lambda_{i}{\gamma_{i}}^{2} \mathrm{for} k=0 \\ \frac{4\eta_{k}\lambda_{i}\varphi_{k}\gamma_{i}}{\beta\left( {\varphi_{k}}^{2}-{\gamma_{i}}^{2} \right)} \mathrm{for} k>0 \end{aligned} \right. (24)$$

$$c_{ik}^{I}=\left\{ \begin{aligned} -\lambda_{i}\gamma_{i} \mathrm{for} k=0 \\ 0 \mathrm{for} k>0 \end{aligned} (25) \right.$$

The exponents are defined,

$$\gamma_{ik}=\left\{ \begin{aligned} \gamma_{i} \mathrm{for} k=0 \\ \varphi_{k} \mathrm{for} k>0 \end{aligned} (26) \right.$$

$\eta_{k}$ and $\varphi_{k}$ are the so-called Padé coefficients which are obtained by first constructing the matrices,

$$\Lambda_{mn}=\frac{\delta_{m,n\pm1}}{\sqrt{\left( 2m+1 \right)\left( 2n+1 \right)}} (27)$$

with $m,n=1,2,\ldots2K+1$, and,

$$\tilde{\Lambda}_{mn}=\frac{\delta_{m,n\pm1}}{\sqrt{\left( 2m+3 \right)\left( 2n+3 \right)}} (28)$$

with $m,n=1,2,\ldots2K$. The eigenvalues of $\Lambda_{mn}$ and $\tilde{\Lambda}_{mn}$ are $\pm2/{\psi_{k}}$ and $\pm2/{\tilde{\psi}_{k}}$ respectively and the Padé coefficients are then defined,

$$\eta_{k}=\frac{\frac{1}{2}R_{K}\prod_{j=1}^{K} \left( {\tilde{\psi}_{j}}^{2}-{\psi_{k}}^{2} \right)}{\prod_{j\neq k}^{K} \left( {\psi_{j}}^{2}-{\psi_{k}}^{2} \right)} (29)$$

where,

$$R_{K}=\frac{1}{4\left( K+1 \right)\left( 2K+3 \right)} (30)$$

Finally, the poles are defined,

$$\varphi_{k}=\frac{\psi_{k}}{\beta} (31)$$

The residual term $\delta C_{iK}\left( t \right)$ is approximated by a Markovian white noise ansatz,

$$\delta C_{iK}\left( t \right)\approx4R_{K}\lambda_{i}\gamma_{i}\beta\delta\left( t \right) (32)$$

One can now construct the HEOM. We do this by replacing the RDO in the Liouville equation with a set of *auxiliary density operators* (ADOs),

$$\frac{d}{dt}\rho_{\mathbf{n}}\left( t \right)=-i\mathcal{L}\rho_{\mathbf{n}}\left( t \right)\mathcal{+D}\rho_{\mathbf{n}}\left( t \right)\mathcal{+K}\rho_{\mathbf{n}}\left( t \right) (33)$$

where the index $\mathbf{n}$ denotes a matrix,

$$\mathbf{n}=\left( \begin{matrix} n_{10} & \cdots& n_{1K} \\ \vdots& \ddots& \vdots\\ n_{N0} & \cdots& n_{NK} \end{matrix} \right) (34)$$

of non-negative integers, $n_{ik}$, for a network of $N$ chromophores ($N=2$ in our case). $\rho_{\mathbf{0}}\left( t \right)$ with index $\boldsymbol{0}=\left\{ 0,\ldots0;\ldots;0,\ldots,0 \right\}$ is simply $\rho(t)$ while all others characterize the evolution of the system-bath correlations. We can now define the dissipation/dephasing term of the Liouville equation as,

$$\mathcal{D}\rho_{\mathbf{n}}\left( t \right)=-\sum_{i=1}^{N} \left( \sum_{k=0}^{K} \gamma_{ik}n_{ik}+\delta\mathcal{R}_{iK} \right)\rho_{\mathbf{n}}\left( t \right)$$

$$+\sum_{i=1}^{N} \sum_{k=0}^{K} \left( \mathcal{B}_{ik}n_{ik}\rho_{\mathbf{n}_{ik}^{-}}+\mathcal{A}_{i}\rho_{\mathbf{n}_{ik}^{+}} \right) (35)$$

where,

$$\mathbf{n}_{ik}^{\pm}=\left( \begin{matrix} n_{10} & \cdots& \begin{matrix} n_{1k} & \cdots& n_{1K} \end{matrix} \\ \vdots& \vdots& \begin{matrix} \vdots& \vdots& \vdots\end{matrix} \\ \begin{matrix} n_{i0} \\ \vdots\\ n_{N0} \end{matrix} & \begin{matrix} \cdots\\ \vdots\\ \cdots\end{matrix} & \begin{matrix} n_{ik\pm1} & \cdots& n_{iK} \\ \vdots& \vdots& \vdots\\ n_{Nk} & \cdots& n_{NK} \end{matrix} \end{matrix} \right) (36)$$

The auxiliary super-operators are defined,

$$\delta\mathcal{R}_{iK}\rho_{\mathbf{n}}\left( t \right)=\frac{4R_{K}\lambda_{i}\gamma_{i}\beta}{2}\left[ \left| \left. i \right\rangle\left\langle i \right. \right|,\left[ \left| \left. i \right\rangle\left\langle i \right. \right|,\rho_{\mathbf{n}}\left( t \right) \right] \right] (37)$$

$$\mathcal{B}_{ik}\rho_{\mathbf{n}}\left( t \right)=i\left( c_{ik}^{R}\left[ \left| \left. i \right\rangle\left\langle i \right. \right|,\rho_{\mathbf{n}}\left( t \right) \right]-ic_{ik}^{I}\left[ \left| \left. i \right\rangle\left\langle i \right. \right|,\rho_{\mathbf{n}}\left( t \right) \right] \right) (38)$$

$$\mathcal{A}_{i}\rho_{\mathbf{n}}\left( t \right)=i\left[ \left| \left. i \right\rangle\left\langle i \right. \right|,\rho_{\mathbf{n}}\left( t \right) \right] (39)$$

The sum of indices,

$$L=\sum_{ik} n_{ik} (40)$$

defines the *tier* of the ADO and we see that the super-operators $\mathcal{A}$ and $\mathcal{B}$ connect ADOs from adjacent tiers. Although the *hierarchy* of ADOs formally extends infinitely it can be truncated at some finite tier without any loss of accuracy so long as the results are converged.

In the description above the site basis was implicitly assumed. However, in this work we adopted the exciton basis, although the distinction becomes blurry outside of the resonance window. In the exciton basis our initial conditions are slightly different, with each exciton state having an initial population proportional to its relative oscillator strength. At resonance, the redistribution of oscillator strength is significant and both excitonic states are optically allowed. As the system deviates from resonance we have a predominantly Chl *a*-like state absorbing and a predominantly Lut-like state being essentially optically forbidden. It is important to remember that the site and exciton bases are entirely equivalent representations of the same system and we are free to transform between the two to give the most intuitive representation of the dynamics. The observable quantity that we calculate, the mean excitation lifetime, is basis-independent.

**References**

^1^Renger T, Marcus RA (2002) On the relation of protein dynamics and exciton relaxation in pigment-protein complexes: an estimation of the spectral density and a theory for the calculation of optical spectra. J. Chem. Phys. 116: 9997-10019

^2^Walla PJ, Linden PA, Ohta K, Fleming GR (2002) Excited-State Kinetics of the Carotenoid S1 State in LHC II and Two-Photon Excitation Spectra of Lutein and β-Carotene in Solution:  Efficient Car S1→Chl Electronic Energy Transfer via Hot S1 States? J. Phys. Chem. A 106:1909 —1916

^3^Ruban AV, Pascal AA, Robert B, Horton P (2001) Configuration and dynamics of xanthophylls in light-harvesting antennae of higher plants. Spectroscopic analysis of isolated light-harvesting complex of photosystem II and thylakoid membranes. J Biol Chem. 276:24862-70

^4^Balevičius Jr. V, Gelzinis A, Abramavicius D, Valkunas L(2013) Excitation Energy Transfer and Quenching in a Heterodimer: Applications to the Carotenoid–Phthalocyanine Dyads J. Phys. Chem. B, 117: 11031-11041

^5^Ishizaki A, Tanimura, Y (2005) Quantum Dynamics of System Strongly Coupled to Low-Temperature Colored Noise Bath: Reduced Hierarchy Equations Approach J. Phys. Soc. Jpn. 74:3131– 3134

^6^Tanimura Y (2006) Stochastic Liouville, Langevin, Fokker–Planck, and Master Equation Approaches to Quantum Dissipative Systems J. Phys. Soc. Jpn. 75: 082001

^7^Hu J, Luo M, Jiang F, Xu RX, Yan YJ (2011) Padé Spectrum Decompositions of Quantum Distribution Functions and Optimal Hierarchical Equations of Motion Construction for Quantum Open Systems. J. Chem. Phys. 134: 244106

**Figures**


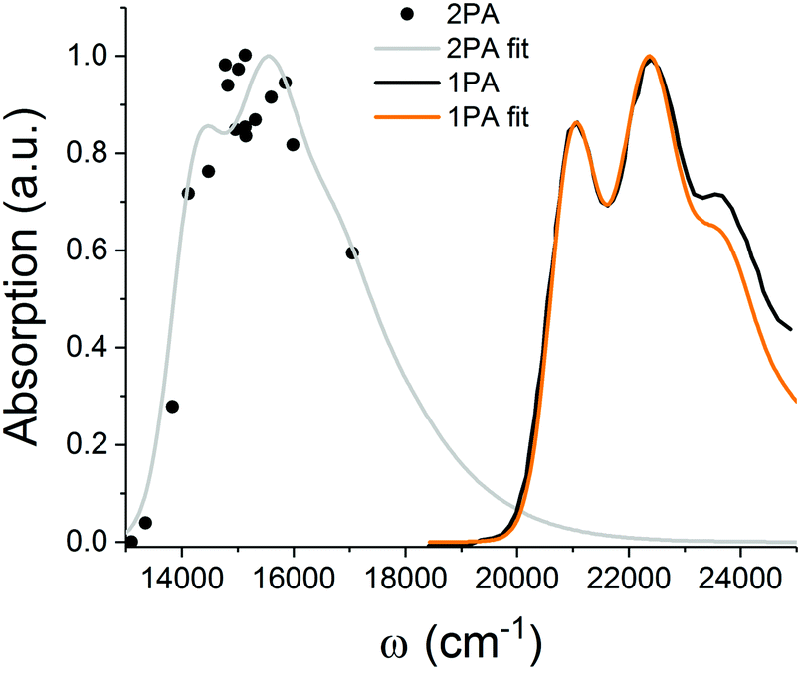


**Supplementary Figure 1:** Visual fitting of the 2-photon absorption (2PA) data for lutein in octanol. This explicitly probes the optically (1-photon) forbidden S_1_ transition. The experimental data points (taken from^2^) are shown as points with the fit shown in grey. For comparative purposes we show a fit (orange line) of a typical lutein 1-photon absorption (1PA) spectrum (black line). 1PA probes the optically allowed S_2_ transition.
